# The complete chloroplast genome of *Notholition macrophyllum*

**DOI:** 10.1080/23802359.2018.1463825

**Published:** 2018-09-10

**Authors:** Juan Li, Haiying Liu, Dengfeng Xie, Songdong Zhou, Xingjin He

**Affiliations:** Key Laboratory of Bio-Resources and Eco-Environment of Ministry of Education, College of Life Sciences, Sichuan University, Chengdu, P. R. China

**Keywords:** Chloroplast, genome, *Notholirion*

## Abstract

*Notholirion macrophyllum* (D. Don) Boiss. (Liliaceae) is a floriferous species naturally distributed in Asia. The complete chloroplast genome sequence of *N. macrophyllum* was generated by de novo assembly using whole genome next generation sequencing data. The complete chloroplast genome of *N. macrophyllum* was 152143 bp in total sequence length and divided into four distinct regions: small single copy region (17913 bp), large single copy region (82222 bp) and a pair of inverted repeat regions (26004 bp). The genome annotation displayed a total of 135 genes, including 82 protein-coding genes, 38 tRNA genes, and 8 rRNA genes. Phylogenetic analysis with 7 Liliaceae species revealed that *N. macrophyllum* was the basal species of tribe Lilieae and was close to *Cardiocrinum giganteum*.

*Notholirion macrophyllum* (D. Don) Boiss. (Liliaceae) is a floriferous herb, which naturally distributes in high altitude region. It wildly grows in quercus forests, grassy slopes and meadows with an altitude in 2800–3400 m (Liang et al. 2000). Its flowers are gorgeous, and similar to lily. *Notholirion macrophyllum* also is used in traditional Chinese medicine (Liang et al. 2000). *Notholirion* Wall. ex Boiss. is a small Asian genus with only 5 species (Liang 1995). There were some researches hypothesized that *Notholirion* is the basal group of tribe Lilieae (Zhou 2008; Gao et al. 2012, 2013). As chloroplast carry maternal genes, it plays an important role in phylogeny reconstruction. However, there are no researches about *Notholirion* chloroplast. In this paper, we first report the complete chloroplast genome of *N. macrophyllum*, which will help in molecular and phylogenetical studies of this plant.

Fresh leaves of *N. macrophyllum* was collected from Daocheng (28°49′33″N 100°27′20″E), Sichuan Province, China. Voucher specimens were deposited in SZ (Sichuan University Herbarium). Total genomic DNA was extracted by Plant Genomic DNA Kit (Tiangen Biotech CO., LTD, Beijing, China). The isolated genomic was manufactured to average 350 bp paired-end(PE) library using Illumina HiSeq platform (Novogene, Beijing, China), and sequenced by Illumina genome analyser (Hiseq PE150). We found *Cardiocrinum giganteum* (Wall.) Makino was the best inference of nuclear genome to contribute for assembly. Contigs, assembled by SOAPdenovo2 (Luo et al. 2012), were sorted and joined into a single-draft sequence using Geneious (Kearse et al. 2012), which compared with the chloroplast sequence of *C. giganteum* as a reference. Gapcloser was used to fill the gapped sites, and the draft sequence was corrected manually by clean read mapping using bowtie2 (Langmead and Salzberg 2012) and Tablet (Milne et al. 2013). The genes in chloroplast genome were predicted using Geneious and corrected manually.

The complete chloroplast genome of *N. macrophyllum* (GenBank accession number MH011354) was 152,143 bp in total sequence length with 37.10% GC contents. Four distinct regions were separated by the complete chloroplast, such as large single copy (LSC) region was 82,222 bp, small single copy (SSC) region was 17,913 bp, and a pair of inverted repeat regions are 26,004 bp in each length. The chloroplast genome detected a total of 135 genes including 82 protein-coding genes, 38 tRNA genes, and 8 rRNA genes.

Aims at clarifying the phylogenetic relationship between *N. macrophyllum* with other Liliaceae species, we generated a maximum-likelihood tree (ML) of 7 species (Figure 1) by MEGA6 (Tamura et al. 2013). There are 6 species, which belongs to tribe Lilieae (Takhtajan 1987), *Amana edulis* (Miq.) Honda pertain to tribe Tulipeae. As shown in Figure 1, *N. macrophyllum* was the basal group of the tribe Lilieae, and close to *C. giganteum*, these results in accordance with early studies (Zhou 2008; Gao et al. 2012, 2013).

**Figure 1.**
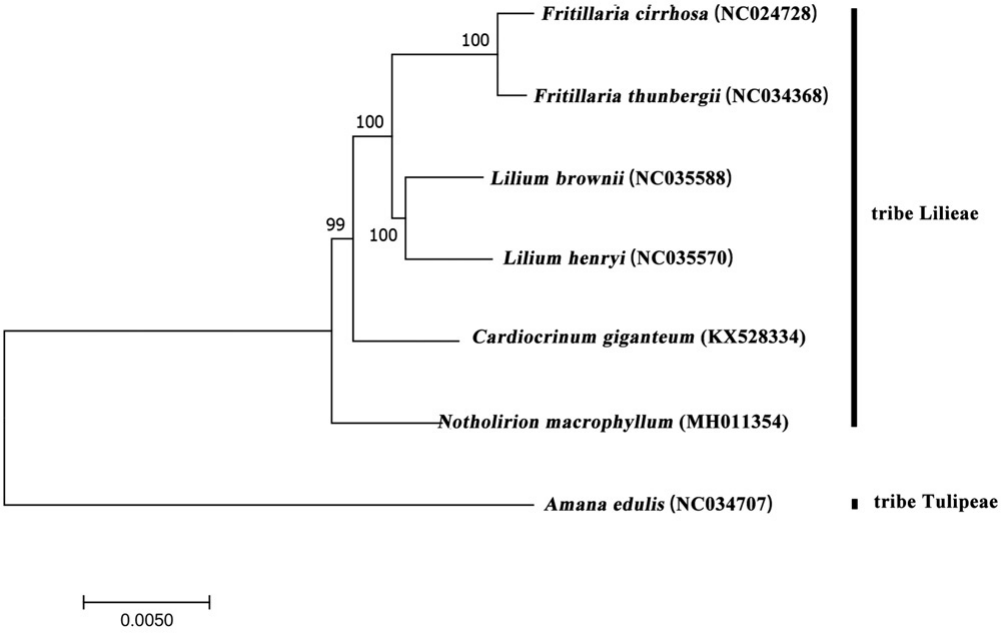
ML phylogenetic tree of *N. macrophyllum* with 7 species was constructed by chloroplast genome sequences. Numbers on the nodes are bootstrap values from 1000 replicates. *Amana edulis* was selected as outgroup.
